# Personnel planning in general practices: development and testing of a skill mix analysis method

**DOI:** 10.1186/1478-4491-12-53

**Published:** 2014-09-18

**Authors:** Juliane von Eitzen-Strassel, Hubertus JM Vrijhoef, Emmy WCC Derckx, Dinny H de Bakker

**Affiliations:** Maastricht University Medical Center, P.O. Box 616, 6200 MD Maastricht, The Netherlands; Scientific Center for Care and Welfare (Tranzo) Tilburg University, P.O. Box 90153, 5000 LE Tilburg, The Netherlands; Health Systems and Policy Saw Swee Hock School of Public Health National University of Singapore, MD3, 16 Medical Drive, Singapore, 117597 Singapore; Foundation for Development of Quality Care in General Practice, P.O. Box 2155, 5600 CD Eindhoven, The Netherlands; NIVEL, Netherlands Institute for Health Services Research, P.O. Box 1568, 3500 BN Utrecht, The Netherlands; Primary Care, Scientific Centre for Care and Welfare (Tranzo), University of Tilburg, P.O. Box 90153, 5000 LE Tilburg, The Netherlands

**Keywords:** Demand, General practice, Skill mix, Supply, Validity

## Abstract

**Background:**

General practitioners (GPs) have to match patients’ demands with the mix of their practice staff’s competencies. However, apart from some general principles, there is little guidance on recruiting new staff. The purpose of this study was to develop and test a method which would allow GPs or practice managers to perform a skill mix analysis which would take into account developments in local demand.

**Methods:**

The method was designed with a stepwise method using different research strategies. Literature review took place to detect available methods that map, predict, or measure patients’ demands or needs and to fill the contents of the skill mix analysis. Focus groups and expert interviews were held both during the design process and in the first test stage. Both secondary data analysis as primary data collection took place to fill the contents of the tool. A pilot study in general practices tested the feasibility of the newly-developed method.

**Results:**

The skill mix analysis contains both a quantitative and a qualitative part which includes the following sections: i) an analysis of the current and the expected future demand; ii) an analysis of the need to adjust skill mix; iii) an overview about the functions of different provider disciplines; and iv) a system to assess the input, assumed or otherwise, of each function concerning the ‘catching up demand’, the connection between supply and demand, and the introduction of new opportunities. The skill mix analysis shows an acceptable face and content validity and appears feasible in practice.

**Conclusions:**

The skill mix analysis method can be used as a basis to analyze and match, systematically, the demand for care and the supply of practice staff.

## Backgrounds

Today’s primary care in the United Kingdom (UK), the Netherlands, and other countries is developing into a service directed towards demand and guided locally [1, 2]. Consequently, the focus of the supply of care will have to be re-directed towards one in which the competences of practice teams are paramount in order to meet the local demand [3]. New professions were introduced in the UK and the Netherlands during the last decades in order to support general practitioners (GPs) in different fields of their work, for example, the practice nurse or the nurse practitioner, who will focus on specific medical and nursing areas. The introduction of these new professions has presented real opportunities for delegating and reshuffling different tasks in Dutch general practice [4]. Other factors, too, have increased the importance of strategic planning for staff in primary care. Most significantly single-handed, mono-disciplinary practices have developed into group practices with several disciplines involved together with ancillary staff (see Table 1 for a statistical summary of general practice in the Netherlands).

**Table 1:** **General practices in the Netherlands** [5, 6]

Number of patients per FTE*	2,350
Practice types	Solo practices: 26%
	Duo practices: 38%
	Group practices (>2 GP’s): 36%
Traditional staff types	GPs
	Practice assistants (0.86 FTE* per GP)
	Practice nurse for chronically ill (0.27 FTE* per GP)
Newer staff types	Psychiatric nurse
	Nurse practitioners
	Physician assistants

*FTE = Full-time equivalent.

GPs have to match patients’ demands with the mix of skills offered by staff working in their practice. However, decisions concerning recruiting new staff are presently based on such general norms as the ratio of full-time equivalent GPs to the area’s population [1, 7–10]. These decisions are often taken *ad hoc* drawn from individual experiences rather than from analyses of factors, such as demand in the practice region, and possible developments in that demand, the current staff performance, or a review of alternative solutions to the skill mix. A more rational approach to making decisions about staff in general practice may contribute to care that is tailored better to the needs of the population and, ultimately, to better care, better accessible care, and/or lower costs [11].

The purpose of this study was, therefore, to develop and test a method to systematically support decisions made about input of staff in general practices in order to match demand with skills. This method is referred to in the remainder of this paper as the ‘skill mix analysis’.

## Methods

The skill mix analysis is developed through a combination of methods following a stepwise approach. These are:

i. A review of the literature in order to make an inventory of methods that map, predict or measure patients’ demand or need, and those which ascertain what is an adequate skill mix in general practices;
ii. Focus group meetings (n = 2) and interviews to assess the usefulness in daily practice of the methods which we identified;
iii. The development of the conceptual version of skill mix analysis;
iv. Focus group meetings and interviews to test its validity and feasibility;
v. An adjustment of the conceptual version to a practical, trial version, based on expert interviews;
vi. Testing the feasibility of the skill mix analysis instrument in a number of general practices;
vii. The release of a final version of the skill mix analysis.

We explain the steps further, grouped into three sections.

### Steps 1–3: The contents of the conceptual version of skill mix analysis

The development of the method of skill mix analysis began with a systematic literature review. Scientific literature was collected which identified methods that map, predict, or measure patients’ demand or need and those which ascertain what is an adequate skill mix in general practices. PubMed, Cochrane Library, and CINAHL were searched for relevant studies and grey literature was searched to retrieve unpublished methods. The methods identified in the systematic literature review were presented in two focus group meetings in order to assess their relevance in daily practice. The experts who were invited included GPs, who were familiar with the reshuffling to tasks (n = 4), university lecturers in professions related to general practice (n = 2), a capacity planner (n = 1), and a researcher (n = 1). The methods identified either measured demand or determined the skill mix on the level of a general practice. Therefore, individual components of these methods were also discussed. The participants were asked to assess which components of the methods are important by discussing: i) What should a skill mix method measure?; ii) On whom should the focus for measuring demand be placed?; iii) Which sources, in particular data, should be used?; iv) How should demand/need and skill mix be determined?; v) How should demand/need and skill mix be illustrated?; vi) How important are the different characteristics of the method?; and vii) How important are other characteristics? In this manner, the various characteristics or components were discussed. Their importance to being part of the method of skill mix was assessed on a Likert-scale from 1 (very low) to 5 (very high). The results of the two focus groups were combined by adding the scores, resulting in a table of characteristics which were most important to include in the skill mix analysis.

The initial aim of this strategy was to combine the components judged to be important for the method. However, we concluded that simply combining the components was not feasible. This was due to the diversity of existing methods and the absence of information regarding the performance of these methods. Alternatively, the relevant components for the method, as derived from the review, and assessed as important by the focus groups, were used as functional requirements from which a first, conceptual version of skill mix analysis was constructed. The approach in the first version was mainly quantitative. Electronic medical record data were derived from the NIVEL Primary care database, a nationally representative network of primary care practices in the Netherlands. This network comprised about 120 practices. Because there were insufficient data concerning practices with nurse practitioners and physician assistants, medical record data were collected for six extra practices of each type. The division of tasks in these practices was analyzed by comparing consultation characteristics, such as the length of consultation and ICPC-coded diagnosis, per staff category and analyzing the workload by calculating consultation rates per full-time equivalent (FTE) member of staff.

### Steps 4–5: Developing the trial version by testing its face validity, content validity, and feasibility of the method

Firstly, a conceptual method was built and visualized using a power point presentation. This version was discussed in a focus group to test its face validity. Participants (n = 8) from the first two focus groups (step 2) attended this meeting. The moderator presented the different components of the method and sought the participants’ views on, for example, the level of interest and the feasibility of collecting the data. The session took two hours and was recorded. A summary of the session was used to analyze the views of the group in order to adjust the conceptual version of the instrument.

A second group of experts was asked to give feedback on the content of the instrument in order to examine the content validity. The experts received the content of the method via email and were asked to write whether they felt the information was up to date, relevant, and complete.

Based on the feedback during this stage, the approach changed from quantitative to combined quantitative and qualitative. To fulfill the qualitative part of the instrument, an additional literature analysis took place of mainly grey literature about, for instance, tasks of different disciplines, characteristics of the training, or salary costs.

This feedback was also used to construct the trial version of the instrument. The trial version was constructed as a web-based internet application programmed with “Personal Home Page Tools”, a server-side scripting language.

### Step 6–7: Testing the final version and releasing it to the public

Ten selected general practices received access to the web-based trial version in July 2012 in order to test the practical feasibility of the skill mix analysis. Three of them did not participate due to their high workload or participation in other research projects. Those participating varied in size and skill mix (Table 2). Testing in these practices took place by providing a link to the trial version of the website. The testers received an identical survey about the feasibility of using the instrument. This survey contained 22 open questions seeking opinion of GPs about the instrument and its individual parts.

**Table 2:** **Test practices**

Number of participating practices	Seven
Average list size	8,300
Practice types	Two solo practices
	Five multidisciplinary health centers
Practice data	2011 and 2013
Number of GPs per practice	Between one and six
Staff composition	Each practice: one or more practice assistants and practice nurses
	Five practices with a psychiatric nurse
	Two practices with a nurse practitioner
	One practice with a physician assistant
Test persons	One GP (three practices)
	A group of GPs (three practices)
	A practice manager (one practice)

In five of the practices, a researcher (JS) observed the application of the instrument by GPs or practice managers using an observation protocol. This was not done in all practices because the researcher may have influenced the testers. Therefore, it was also important to have test results from practices where there was no researcher present. For each stage, as discerned in the instrument, the researcher’s observations were written down. Furthermore, the time needed to use the skill mix analysis was written down (JS).

Feedback from the test was used to adjust the web-based version of the skill mix analysis. Questions and problems that occurred during the trial were clarified in the final version. The method was then published on a website.

## Results and discussion

The results are described according to the seven steps for the development of the skill mix analysis.

### Steps 1–3: The development of the conceptual version

#### 1. Reviewing the literature

The review of the literature identified 27 methods; 22 support the process of measuring demand or need, and 5 the process for determining the skill mix [10]. The findings were sub-divided into three categories, indicating whether the method measures need (for example [12, 13]), confronts demand and supply (for example [14, 15]), or determines skill mix (for example [16, 17]). None of the methods identified systematically aligned demand and skill mix in general practice. From this review, it was concluded that a ready-made instrument to translate demand into an adequate skill mix within general practices is lacking. However, elements from existing methods could be used to construct such an instrument.

#### 2. Focus group meetings for initial assessment of the usefulness in daily practice of the methods identified

The results of the literature review were summarized showing the characteristics of all the methods identified (n = 42). The focus group members scored the characteristics individually. The characteristics were grouped according to importance as very important (experts scored 4 or 5, n = 5), important (expert scored between 3 and 5, n = 15), less important (score between 2 and 4, n = 3), or not important (score 1 of 2, n = 0). There was no consensus (n = 19) for several characteristics (Table 3).

**Table 3:** **Characteristics to be included in the skill mix analysis**

(1) What should a skill mix method measure?	Needs from patient’s perspective (3–5)
	Needs from professional’s perspective (3–5)
	Community’s own perspective of its needs (3–5)
	Present demand (n.c.)
	Future demand (3–5)
	Present capacity (n.c.)
	Confront demand and supply (3–5)
	Forecast effect of changes in patient demand (4/5)
	Whether skill mix is generally the solution to health delivery problems (3–5)
	Whether there is balance between patients care demand/needs and professionals time resources (3–5)
	Task distribution (4/5)
	Task overlap among primary care team members (n.c.)
	Workload (n.c.)
	Importance of each job task for each professional (perspective of the professional) (2–4)
	Time spent per task per professional (2–4)
	Training needs of health care professionals (n.c.)
(2) On whom should the focus for measuring demand be placed?	Patients of a practice/health care center (n.c.)
	Population (n.c.)
	Community (n.c.)
	Practice and community (3–5)
	Special patients groups (elderly, chronically ill, etc.) (n.c.)
(3) Which sources, in particular data, should be used?	Already available data (i.e., medical records) (3–5)
	Collect additional data (qualitative or quantitative) (4/5)
	Knowledge of the primary health care team (n.c.)
(4) How should demand/need and skill mix be determined?	Based on simplified classification areas of demand/need (e.g., planning or coordinating care, prescribing, guidance in care, etc.) or skill mix (e.g., define core tasks) (3–5)
	Based on a very detailed overview of demand/need and skill mix (n.c.)
5) How should demand/need and skill mix be illustrated?	Purely descriptive/reporting (numbers) (n.c.)
	Visual overview (e.g., create simple analytic maps, baseline snapshot of practice’s patient population demographics) (3–5)
(6) How important are the particular characteristics of the method?	Expenditure of time (quickly applicable) (3–5)
	Practicability (simple to apply) (3–5)
	Costs for applying the instrument (n.c.)
7) How important is it that the method enables to:	Identify strengths and weaknesses within a multidisciplinary primary health care team (4/5)
	Conduct a comparison between practices (comparative approach) (n.c.)
	Forecast demand (n.c.)
	Identify health need priorities (n.c.)
	Identify health inequalities (2–4)
	Interpret practice data (3–5)
	Manage workload (n.c.)
	Support planning of staffing needs (competencies) (4/5)
	Forecast amount of staff required (personnel planning) (n.c.)
	Conduct long-term strategic planning (3–5)
	Conduct a comprehensive environmental analysis (identify risk factors and causes of ill health, accessibility, efficiency, etc.) (n.c.)

Explanation of scores:

Very important (4 or 5, n = 5); Important (between 3 and 5, n = 15); Less important (between 2 and 4, n = 3); Not important (1 of 2, n = 0); (n.c.) = No consensus (n.o consensus: between 1 and 5, n = 19).

Most characteristics were rated as of sufficient relevance to be part of the instrument. This confirmed, on the one hand, its relevance, but, on the other, made combining of the differing characteristics challenging. The results of the expert assessment were used to indicate which characteristics are important for inclusion in the new method (see Table 4 for themes of the focus groups).

**Table 4:** **Themes focus groups I and II**

Focus group I	Additions to the identified literature/projects/methods and discussion of:
	– the relevance of a skill mix method
	– todays personal decision in practice
	– evaluation of the questionnaire
Focus group II	Feedback on the current draft of the skill mix method:
	– discussion of the content: at this point task clusters of patient-related and other tasks per profession
	– usefulness of the current method for practice
	– potential improvements and additions

#### 3. The development of a conceptual method

The components deemed relevant by the review, and assessed important by the focus groups, were first taken together in the initial conceptual version of the skill mix analysis. There was general agreement that a web-based application would be the most practical solution to make the instrument available to the target group. A paper model, using example data, was constructed in order to achieve ‘functional’ specifications for the programmer of the application. The approach was mainly quantitative. It comprised four components: i) the method to measure current demand; ii) current supply; iii) future demand; and iv) future supply.

### Steps 4–5: Developing the trial version by testing the method’s face validity and content validity

#### Focus group meeting to examine the validity of the content

**Figure 1:**
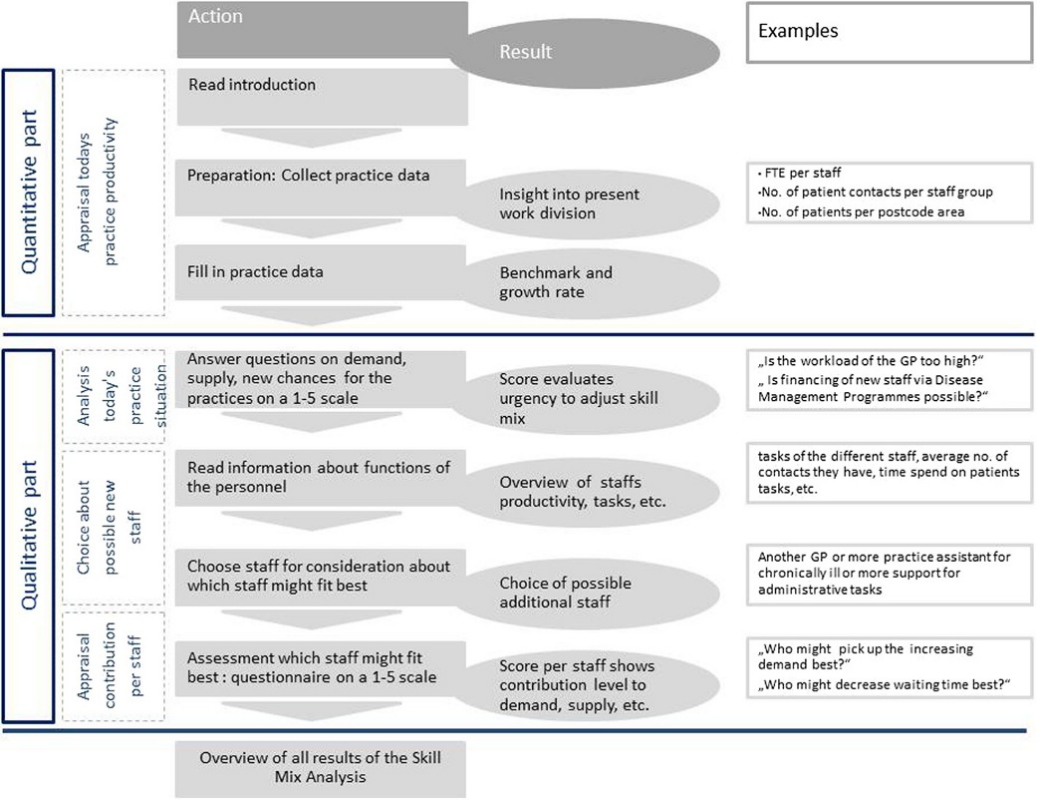
**Overview of the skill mix instrument.**

The focus group feedback was used to adjust the conceptual version. This showed that the data collection might be difficult for some practices because it was not always possible to generate a distribution of consultations over a variety of members of staff. Therefore, the systematic overview of task division might not always be possible. Problems emerged over how one would gain insight into the amount of time each member of staff invested in activity orientated towards the patient, as opposed to time spent on overhead activity.Based on the feedback from the focus group, it was concluded that the approach cannot be exclusively quantitative. A new version was developed with a much smaller quantitative part with data for three out of the four original components (current demand, current supply, and future demand). New, qualitative parts were added. An overview of the adjusted method is given in Figure 1.

### A description of the skill mix analysis

**Figure 2:**
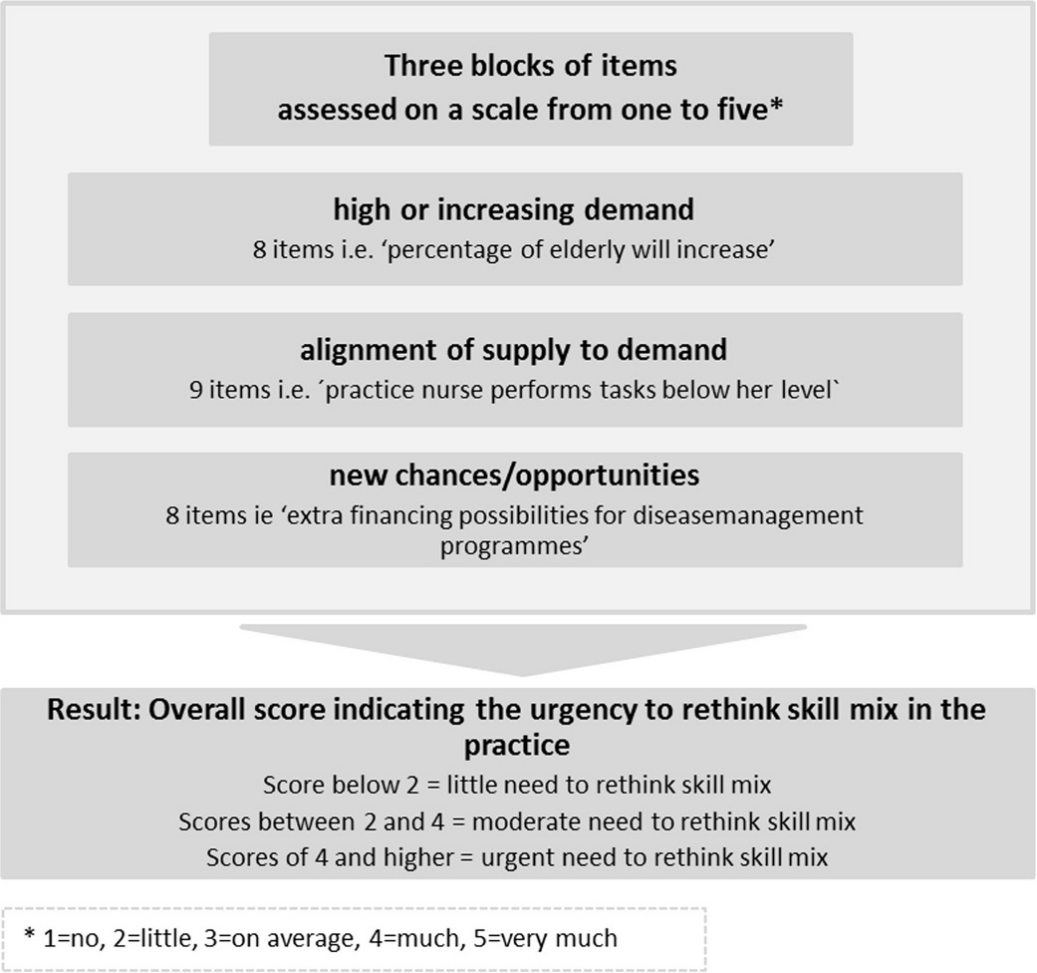
**Qualitative part of skill mix analysis.**

The web-based skill mix analysis contains both a quantitative and a qualitative parts which include the following sections: i) a quantitative analysis of the current, and expected, future demand; ii) a qualitative analysis of the perceived need to adjust skill mix; iii) an overview, with information on staff types; and iv) a qualitative assessment of the contribution of these staff members to the perceived skill mix problems.In the first, quantitative part, the user needs to fill in practice data such as the number of patient contacts per staff group. These data are usually extracted from the general practice medical record system (HIS-system). Subsequently, the skill mix analysis provides a benchmark comparing the productivity of the practice with the national average and a growth percentage for the expected future demand of the practice. The information derived from the first part is used as input for the following qualitative part. Practice staff was then asked to draw conclusions about the current situation (Figure 2).

Subsequently, the user receives information about different professions: GPs, assistants, practice nurses, psychiatric nurses, nurse practitioners, and physician assistants. Information is given, per profession, on the content of the staff’s education, tasks generally performed by this profession, average productivity figures, time use data, and salary guidelines. Finally, other considerations derived from the literature, which can play a role when hiring one of these professionals, are given. The information for this part was partly derived from the medical record data and partly from additional literature analysis.

The last step is, once more, a qualitative assessment with the same items as used in step 2. The practice makes its own appraisal of the contribution of selected professions in order to solve the skill mix issues of the practice. Based on a scoring system (again 1 to 5), the practice can test what kind of staff mix would fit best with the demands of the practice.

### 5. Adjusting the conceptual version of the skill mix analysis

#### Expert interviews to examine the validity of the content

Feedback from the expert interviews on the content of the new conceptual version gave rise to small changes such as adding up to date information concerning the type of tasks each profession is able to do.

The method is tested within general practices through a questionnaire in order to examine the methods feasibility.

### 6. General test results

The method’s basic idea was judged to be useful. One of the testers said that “*the application helps to think about things which play a role in the decision-making process*”. Another tester mentioned that the method “*helps to get insight in own practice data and to come to an estimation of which staff would support the practice best*”.

The different steps of the method can play a role in decisions made about staff and offer logical steps towards decision making. The structure of the method and the navigation of the website were judged to be logical and comprehensible. The use of the instrument took about one hour for each test. A further hour was planned for the questionnaires. Little help was needed beforehand with collecting the necessary data. In four cases, the method was judged to be supportive in that it gave a direction for personnel decisions. The users seemed to receive a good overview about what aspects might play an important role in making decisions about staff.

Benchmark data, for comparison with the practice’s own data, were named as one of the most supportive parts of the method. However, it must be clarified in advance which numbers need to be collected and a more precise description needs to be added about the exact meaning of the data included in the benchmark.

In one case, the result of the assessment indicated that a practice nurse should be appointed for chronically ill patients. However, according to the practice, there was no need for more support in this area. Apart from this exception, the results of the final advice of the method broadly matched the practice’s own expectations with regard to which staff would support the type of demand in the practice the most.

### The scores of the test practices

**Figure 3:**
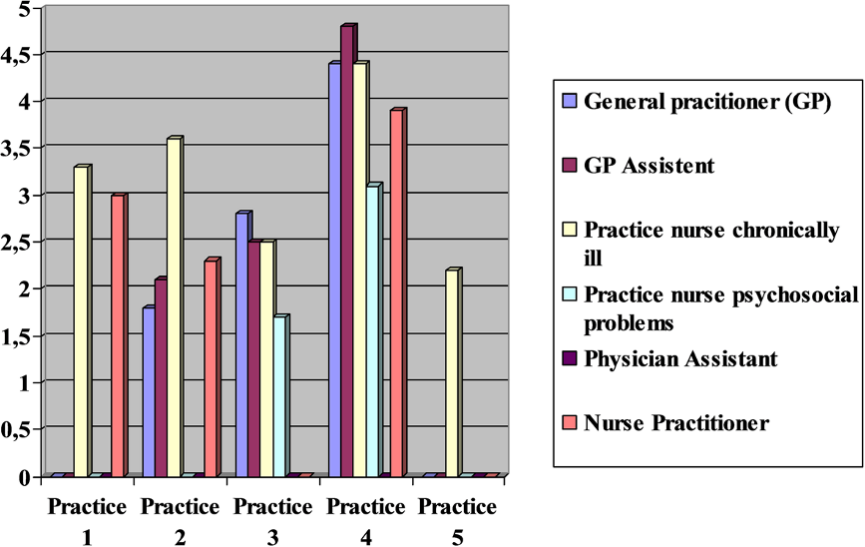
**Scores of contribution staff types in solving skill mix problems for testing practices.**

The assessment of the current situation within the practice resulted in a variety of scores relating to the urgency to rethink skill mix. Four practices scored between 1 and 2, indicating little need to adjust skill mix. Three practices scored between 2 and 4, a moderate to urgent need to rethink skill mix. An urgent need to rethink skill mix (score of 4 to 5) was not scored.Figure 3 shows the disciplines for which the practices performed the skill mix analysis. The resulting scores indicate the degree to which appointing a staff member for that discipline would improve skill mix in the sense that supply and demand would be better aligned.

As an example, practice 1’s current skill mix consists of 3.9 FTE GP, 5.1 FTE GP assistant, 1.1 FTE practice nurse for the chronically ill, 0.4 FTE psychology practice nurse, and 0.8 FTE nurse practitioner. The practice scored 2.6, indicating little to moderate need to rethink skill mix. The evaluation of the demand in this practice showed, amongst others, that the demand is higher than on average, the workload of the GPs is considered too high, and that there is potential for financing care programs. As a result of the skill mix analysis, a practice nurse for the chronically ill and a nurse practitioner would support the demand of the practice best due to the type of demand and the analysis of which type of staff would address this demand best.

In all practices, the practice nurse caring for its chronically ill patients was selected in order to assess whether they could offer support in meeting demand and offer the necessary provision of care for the practice. In the Netherlands, practice nurses caring for the chronically ill are authorized to conduct follow-up consultations with their patients according to medical guidelines. In three practices, the physician assistant, the nurse practitioner, the GP and the GP’s assistant were selected for an assessment. The assessments did not result in a score under 1.7 (1 = no contribution to solving the skill mix issues). The lowest score was awarded to the practice nurse caring for patients with psychological problems in practice 3. This indicated that this member of staff contributed the least to solving the practice’s supply and demand issues. The highest score was 4.8 (5 = a considerable contribution to solving the skill mix issues). This score was awarded by practice 4 to the practice assistant. This indicates that more practice assistants and administrative support are needed. There was, however, a large variation both within and between practice scores.

### Comments and problems during the test

General comments concerned the way the benchmark data were used. The data collection was in some cases quite time consuming, taking up to two hours. The data collection was easier in some practices than in others due to different practice data systems and possibilities of managing them.

Furthermore, the data about the number of contacts for the benchmark could be counted in different ways. In the current method, each type of patient contact counted as one contact irrespective of the type or length of consultation. Some staff members, for example practice nurses, have consistently longer consultation times than others. Furthermore, the registration of a patient contact per staff is not always reliable. This is because, when treatment rooms are changed during the day, staff may record, not only under their own initials, but also under the initials of a colleague.

Another difficulty with figures for productivity was that in some practices GP contacts cannot be distinguished from assistant contacts. This is because both types of staff play a role in a single patient consultation. However, this is not registered.

In two cases, the overall outcome of the skill mix analysis was different from what the users expected. The results of the analysis are based on the practice’s own estimates. However, the users expected the method to give concrete advice, based on their practice data, about which staff was needed.

In one practice, the overall score of the method was judged to be difficult to interpret for concrete decisions. The GP in this practice suggested adding a section to the method to make it more concrete in relation to giving feedback about the current situation in the practice.

### Step 7: Adjusting the second version and offering the public access to the skill mix analysis

#### Final version and its release

The problems concerning clarity and the degree of explicitness were adjusted according to the feedback. Missing items were added where possible, for example, concerning costs in the qualitative analysis or whether enough rooms are available to appoint new staff. The feedback of the practices was used to adjust the method in terms of small changes as updates and additions to the information given in the model (e.g., what does the abbreviation mean?), technical questions (is it possible to save the data on an own account?), and user friendliness (can I print my results?). A final version was developed and published online (http://skillmix.nivel.nl/skill/index).

## Discussion

The aim of the study was to develop and test a method to support decision making about staff among general practices in relation to matching demand with skills. This study presents a web-based internet method combining a quantitative and a qualitative approach. This first version of the skill mix analysis was perceived as useful in guiding the decision-making process as it covers all essential steps in making decisions about staff.

The method offers a first attempt to strengthen the awareness of GPs about what kind of factors need to be taken into consideration when deciding on which staff to enroll. The method can be used to develop a vision on human resources and could support a fresh approach to the delegation of tasks and staffing.

The development of the instrument proved to be complex. It was challenging to find a starting point due to a lack of a comparable method. This might have allowed us to begin to investigate decisions about how staff can be supported systematically [11]. Another challenge to develop such a method was the lack of reliable data on which decisions about staff can be based. The initial idea was to make an overview of all the different tasks performed by different staff based on the demand. However, allocating tasks to different staff was not feasible due to missing data and time consuming procedures for data collection.

The current division of tasks and the demands of general practice need first to be analyzed before one can discover the advantages in changing the skill mix. However, there are shortcomings in the data collection required to achieve a clear picture about the current division of tasks within a practice. For example, based on electronic medical records (EMR) data, it is difficult to show the current division of tasks within a practice. The EMR data are sometimes difficult to interpret. Sometimes staff do not register under their own identification, which makes a comparison of productivity per employee impossible [18]. Furthermore, there is a lack of information about the use of time in patient contacts per staff. There are no data yet which give an overview of the time taken to complete different tasks, or rather to differentiate between tasks that are, and those that are not, directed towards the patient. In particular, national productivity figures relating to the more recently launched professional roles, such as the physician assistant and the nurse practitioner, are missing because they have only recently become involved in general practice and are not represented nationally [19].

The data issues need to be addressed in order to further develop the model and to consider ‘task shifting’. As the model is only considering the skill mix capacity required based on current work, rather than full skill mix shifting of patients or treatments across the skill mix spectrum for the practice, the model can be developed further. For example, by routinely collecting good data. Better data collection could be done, on the one hand, by the practices themselves (e.g., more precise data registration under each staff initials) and more general research, on the other (e.g., about time spent per patient contact). Practices may be followed up in future research by applying the skill mix analysis again and compare the evaluation with earlier results.

The calculation of the future demand in the skill mix analysis is based on the calculations of a webtool to analyze demand for and supply of primary care. This method calculates expected present and future demand by applying medical record data to a demographic data of neighborhoods, using a spatial micro-simulation approach [9]. The tool gives an insight into changing demand on a neighborhood level and can be used as an extra help for users of skill mix analysis.

Evidence on the effects of changing skill mix is crucial for the development of an instrument such as skill mix analysis. This evidence is, however, scarce. Of course such evidence would be of great interest for the decision-making process of practice managers [20].

Research shows that changes in skill mix, that is introducing new providers, will, in itself, affect demand. The introduction of a nurse practitioner in a general practice might not only substitute consultations but also generate extra or longer consultations. At the same time, the GP might fill the extra time he or she receives with extra, longer, or more complex consultations. Furthermore, introducing new providers will take more time to co-ordinate [21]. Whether the extra or longer consultations are ‘overutilization’ or whether there was ‘underutilization’ in the past is difficult to establish, but the dynamic interplay between supply and demand is obvious. Data on experiments with an alternative skill mix are needed to simulate alternative scenarios. However, this type of research is difficult to conduct as changing skill mix, in itself, might already change demand. One example is the introduction of practice nurses who specifically care for chronically ill patients and perform check-ups which in turn lead to more consultations since these were not undertaken systematically by the GP. Moreover, the consultation time of the practice nurse was, on average, longer than the GP consultations. Therefore, the total consultation time increased even further [22]. This was the price paid for better adherence to the guideline and thus quality of care [23].

The website has been published recently (October 2013). The number of users is now up to 100. So far, there is no information on the effects of the use of the website. Our first impressions suggest that the people using the method tend to be in a position to make decisions such as practice managers of health centers and senior partners in GP practices. They are interested in a consideration of factors on which skill mix decisions can be based. The relevance and willingness to use the instrument is greater when there is some kind of dynamics in demand or supply such as meeting a fast growing demand in new residential areas or decreasing populations in rural areas where it is difficult to attract new GPs. GPs retiring, or workloads of GPs perceived as high, are other reasons to rethink skill mix. Reductions in staff and possible loss of jobs are, in the Dutch context, not real issues limiting the willingness to use the instrument, because demand for primary care is generally increasing. Concerns among GPs about shifting tasks from GPs to nurses might be a factor limiting the utilization of the instrument. On the other hand, the instrument contributes to raising awareness that there are more skill mix solutions than simply bringing in more doctors.

### The strengths and limitations of the study

The study benefited from the involvement of several methods. The development of the method was preceded by a comprehensive literature review, interviews with experts in the field, and the involvement of researchers who are familiar with the development of methods to analyze supply and demand. A pilot test was conducted in a practice by a researcher who observed the problems which occurred and adjusted them in the first version of the method. In this way, the development of the method took place in an iterative way providing several opportunities to re-assess former steps in the process.

Due to missing productivity data about newer professionals (physician assistant and nurse practitioner), practice data were collected and questionnaires were conducted to gain a first insight into their productivity. However, these data are not comprehensive due to the relatively low number of practices involved. Furthermore, the practices which have participated in the research are not nationally representative as they are more innovative than average practices and do generally think more about changes to improve the practice performance.

The existing method is limited in that no picture of the optimal skill mix for the practice, given the characteristics of local demand, is given. Given the limitations of the data and the lack of evidence of the effects of alternative skill mix solutions, such an algorithmic approach is impossible. The practice manager or GPs own assessments of the situation are an essential part of the instrument.

The beauty of this method is that it stimulates a more systematic process of thinking about skill mix issues, instead of the existing *ad hoc* process of making these decisions. A limitation concerning the testing of the method is that observational bias might have occurred during the evaluation of the method as the presence of the researcher might have influenced the judgment or comments of the testers.

## Conclusions

The web-based skill mix analysis gives insight into the factors determining a general practice’s decision on staff. General practices can use the method as a starting point to analyze their supply and demand and to review what type of staff might be best suited to the type of demand in their practice.

## Abbreviations

FTE: Full-time equivalent
GPs: General practitioners.
